# Laser-sculptured ultrathin transition metal carbide layers for energy storage and energy harvesting applications

**DOI:** 10.1038/s41467-019-10999-z

**Published:** 2019-07-15

**Authors:** Xining Zang, Cuiying Jian, Taishan Zhu, Zheng Fan, Wanlin Wang, Minsong Wei, Buxuan Li, Mateo Follmar Diaz, Paul Ashby, Zhengmao Lu, Yao Chu, Zizhao Wang, Xinrui Ding, Yingxi Xie, Juhong Chen, J. Nathan Hohman, Mohan Sanghadasa, Jeffrey C. Grossman, Liwei Lin

**Affiliations:** 1 Department of Materials Science and Engineering, Massachusetts Institute of Technology, Cambridge, MA 02139 USA; 2 Mechanical Engineering & Berkeley Sensor and Actuator Center, University of California Berkley, Berkeley, CA 94704 USA; 3 Department of Engineering Technology, University of Houston, Houston, TX 77204 USA; 4 College of Electronic Science and Technology, Shenzhen University, 518060 Shenzhen, China; 5 Micro and Nanosystems, D-MAVT, ETHZ, Zürich, CH – 8092 Switzerland; 6 Molecular Foundry, Lawrence Berkeley National Lab, Berkeley, CA 94720 USA; 7 School of Engineering and Applied Sciences, Harvard University, Cambridge, MA 02138 USA; 8 Aviation and Missile Center, U.S. Army Combat Capabilities Development Command, Redstone Arsenal, AL 35898 USA

**Keywords:** Electrocatalysis, Design, synthesis and processing

## Abstract

Ultrathin transition metal carbides with high capacity, high surface area, and high conductivity are a promising family of materials for applications from energy storage to catalysis. However, large-scale, cost-effective, and precursor-free methods to prepare ultrathin carbides are lacking. Here, we demonstrate a direct pattern method to manufacture ultrathin carbides (MoC_x_, WC_x_, and CoC_x_) on versatile substrates using a CO_2_ laser. The laser-sculptured polycrystalline carbides (macroporous, ~10–20 nm wall thickness, ~10 nm crystallinity) show high energy storage capability, hierarchical porous structure, and higher thermal resilience than MXenes and other laser-ablated carbon materials. A flexible supercapacitor made of MoC_x_ demonstrates a wide temperature range (−50 to 300 °C). Furthermore, the sculptured microstructures endow the carbide network with enhanced visible light absorption, providing high solar energy harvesting efficiency (~72 %) for steam generation. The laser-based, scalable, resilient, and low-cost manufacturing process presents an approach for construction of carbides and their subsequent applications.

## Abstract (web summary)

Transition metal carbides are attractive for electrochemical energy storage and catalysis, but cost effective preparation on a large scale is challenging. Here the authors use a direct pattern method to fabricate transition metal carbides for supercapacitors and solar energy harvesting for steam generation.

## Introduction

Transition metal carbides (TMCs) have unique characteristics such as low resistivity (metallic), high melting temperature, and high electrochemical activities for energy storage and catalysis^1^. Dimensional reduction of bulk TMCs to 0-, 1-, or 2-dimensional nanostructures (thickness vs lateral size < 1 %)^2,3^ has attracted interest as a means to provide further control over a range of properties as well as to introduce new functionality^4–6^. TMCs in particular have gained recent attention due to their intriguing physical, electrical, and catalytical properties. In general, the state-of-the-art fabrication process for ultrathin 2D-TMCs (MXenes)^7–10^ involves high temperatures (up to 1600 °C)^11^ to generate the MAX phase (M_n+1_AX_n_, where M is a transition metal, A is a group 12–16 element, and X is C or N) precursor and then a hydrofluoric (HF) acid etching process to remove the A layer to form M_n+1_X_n_T_x_ (T: OH, OOH and other surface terminations)^9,12–14^. Fluoride-free methods for preparing ultrathin TMCs have been reported, notably chemical vapor deposition (CVD) on metal foils or graphene but the yield is extremely low^3,15,16^. Therefore, development of a robust, mild temperature, versatile fabrication, and precursor independent method for ultrathin TMCs is required to enlarge the family of ultrathin-TMCs, expand the understanding of their properties, and enable cost-effective large-scale synthesis. Meanwhile, the etched MXenes sheets are terminated by rich surface functional groups such as –OH and –COOH, which could change the properties of intrinsic carbide. For example, Ti_3_C_2_T_x_ decay at 200 °C due to the collapse of surface groups^17^ while the Ti_3_C_2_ shall survive up to 1000 °C. Synthesis of “purer” TMC beyond MXenes will empower researchers with wide opportunities to study their intrinsic physics and chemistry. Beyond the synthesis of the materials, their facile integration into devices represents another key challenge in realizing the potential of TMCs for applications. Devices made from chemically synthesized TMCs require further lithography steps; instead, a direct patterning method would enable roll-to-roll manufacturing of host of potential applications from energy storage devices^18^ to wireless communication^19^.

**Fig. 1:**
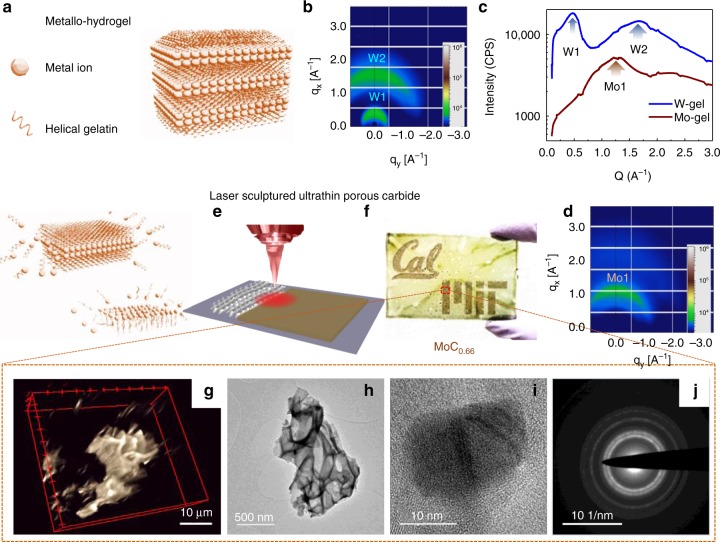
Schematic of laser-sculptured ultrathin transition metal carbides. **a** Process for producing laser-induced transition metal carbides. Helical polymer gelatin mediated transition metal ions (metallo-hydrogel) are used to form a layer-by-layer structure. **b**, **c** Grazing incident wide angle X-ray scattering (GIWAX) characterizations of metallo-gels. **b** GIWAX of W-gelatin made dissolved in NMP (N-Methyl-2-pyrrolidone)^32^. **c** Integration of GIWAX scattering data of Mo-gel and W-gelatin-NMP. **d** GIWAX of Mo-gelatin template as an example to show that gelatin constrains the metal ions laterally between the layers^32^. The Mo1, W1, and W2 peaks correspond to peaks in the GIWAX scattering in **b** and **d** and show how the ion type and solvent in the template greatly affect the assembled layer distributions. **e** IR laser ablation generates highly porous structures with interconnected curved surfaces, for which the “wall” thickness reaches down to the nanometer scale. **f** Using the molybdenum ion as an example, carbide features of “MIT” and “Cal” are engraved on a yellowish transparent thin film (~8 μm in thickness) spin-coated onto a glass substrate with a computer aided design software. **g** Confocal image of 3D tomography or a selected area of laser-sculptured carbide. **h** TEM image of laser synthesized MoC_x_. **i** High resolution TEM images of MoC_x_ showing the sizes of nanocrystals are around 10 nm. **j** Selected area diffraction of MoC_x_. The diffraction ring indicates a polycrystalline structure within the carbide layer

Herein, we demonstrate a versatile process to fabricate transition metal carbides in the form of ultrathin flakes with few-nanometer thickness. The approach utilizes laser ablation of a lamellar hydrogel/metal-ion matrix (metallo-hydrogel). Gelatin, commonly found in jelly desserts, can undergo a self-assembly process to form membranes in a layer-by-layer ordering^20–22^. By selecting carbide-forming metal ions (Mo^5+^, W^6+^, and Co^2+^), a nanostructured hydrogel can be prepared that is ablated for subsequent conversion into TMCs that retain their supramolecular layer-by-layer structure^23,24^. When a hydrogel with embedded transition metal ions is heated, self-accumulation of polymeric phases of metal carbide degradation products occurs at the hydrogel air/liquid interface in a process described by the Buoyancy–Marangoni effect (Fig. 1a–d)^25–27^. The self-assembly and ordering of gelatin can therefore macroscopically arrange precursors into structures that can be collapsed into dimensionally conserved nanostructures^28,29^. When ablated by a CO_2_ laser (Fig. 1e), metallo-gel absorbs IR energy which generates high temperatures (over 2000K, supporting information) within a rapid uptake time (sub millisecond timescale)^30^. Residual solvent and carbon source from the gel “explosively” vaporizes and reacts with metal ions to form carbide structures (MoC_x_, WC_x_, and CoC_x_) with macroporous (50–1000 nm pore size^31,32^). The metallo-hydrogel can be easily spin coated onto a variety of substrates including glass (Supplementary Fig. 1). A common commercial cutter (VLS 2.30, Universal Laser) can directly pattern features (Fig. 1f) and integrate electrodes for microdevices with resolution of 25 microns. The laser-patterned carbide, using MoC_x_ as an example, performs as an energy storage interdigit supercapacitor electrode having a wide operational temperature range (−50 °C to 300 °C in electrolyte). Furthermore, the single laser step not only produce ultrathin-TMC, but also pop up the ultrathin sheets into 3D microstructure with interconnected surface. As-sculptured structure provide more tunability in optical, electrical, and mechanical properties. For example, a membrane made of surface curved MoC_x_ can localize light with the to enhance solar energy harvesting (72% energy efficiency, evaporation total enthalpy over incident solar illumination energy). The exceptional thermal resilience of TMCs could enable a range of other applications such as carbide-based supercapacitors or solar-steam generation membranes operating in harsh environments.

## Results

### Materials characterizations and properties

The local high-temperature generated rapidly by the IR laser pulse^33^ (Table S1), enables an instantaneously high carbon uptake and induces higher energy phases (Supplementary Fig. 2, Supplementary Fig. 3)^34^. Within 30 μs the accumulated temperature can reach up to 2000 K in the Mo-gel thin film (Supplementary Fig. 3a–c) with a projected power of 4 W, using Mo-gel (2 molal, 60% wt) as an example. The rapid initial uptake leads to the formation of the carbon-deficient, metastable phase α-MoC_x_ instead of the thermodynamically stable Mo-C (α/β-Mo_2_C) phases (Supplementary Fig. 2). A simplified heat transfer model was employed to estimate the temperature profile (Supporting Information), with approximate timescale within the range of µs to ms^35^. The laser energy input produces this high energy α-MoC_x_ (0 < x < 1) phase of MoC_x_ (Supplementary Fig. 2), which has a threshold temperature of 1928 K in the phase diagram The product of α-MoC_x_/C has a much higher Gibbs free energy than the common α-Mo_2_C and β-Mo_2_C^36–38^ and the 2D-Mo_2_C achieved by CVD^3,15,16^. Chemically exfoliated Mo_2_CT_x_^2,39^ corresponds to β-Mo_2_C, which is a lower energy phase compared to α-MoC_x_. Confocal optical mapping of the laser-induced carbide (using MoC_x_ as an example), shows three-dimensional curved porous structure (Fig. 1g), and the slices of different confuse plane shows different cross section in the 3D tomography (Supplementary Fig. 4). Scanning electron microscopy (SEM) and transmission electron microscopy (TEM) images show that the broken-down sheets of the porous structure have a thickness of 10–20 nm and are comprised of randomly orientated nanocrystals (Fig. 1h–j, Supplementary Fig. 4d–e). In comparison, for the cases of Mo-PEO and Mo-PVP, the temperature is much lower due to their lower IR absorption coefficients^18^, and the laser carbonized products are not recognized as any carbide phase (Supplementary Fig. 3d, e). Two other carbide phases β-W_2_C/W and Co_2_C/Co (Table 1, Supplementary Fig. 5), were not exfoliated from the corresponding MAX phases^40^.

**Table 1:** Summary of the composition of various hydrogels and their obtained products with laser ablation

Salt Precursor	Solvent	Polymer media	Product
MoCl_5_ (2 m)	DI water	Gelatin (>30 wt%)	**MoC**_**x**_
MoCl_5_ (2 m)	DI water	Gelatin (1 wt%)	MoO_2_
MoCl_5_ (2 m)	DI water	Gelatin (5–10 wt%)	MoO_2_ + Mo_2_C
MoCl_5_ (2 m)	DI water	PVP	NA
MoCl_5_ (2 m)	DI water	PEO	NA
WCl_6_ (1 m)	NMP	Gelatin	W_2_C + W
CoSO_4_(2 m)	DI water	Gelatin	Co_2_C + CoC_x_
NiSO_4_(2 m)	DI water	Gelatin	Ni + NiO_x_ + NiC_x_
FeCl_3_ (2 m)	DI water	Gelatin	Fe + FeO_x_ + FeC_x_
Zr(NO_3_)_4_ (2 m)	DI water	Gelatin	ZrC + ZrO_2_
Zn(NO_3_)_2_ (2 m)	DI water	Gelatin	NA
Tetrabutyl Titanate	NMP	Gelatin (<5 wt%)	TiC + TiO_2_

Further technical details regarding the laser power, spot size, temperature profile and product are discussed in supporting information, Supplementary Fig. 6. A 2 W laser with a scan speed of 200 mm s^−1^ results in the smallest amount of amorphous carbon on Mo-gel (2 m, 60 wt%), and decent conductivity of ~3.2 S cm^−1^ for an 8-μm-thick spin-coated Mo-gel thin film (Fig. 1d), and is used as the standard protocol to pattern carbide devices in the rest of this paper. Electrical conductivities of laser-ablated carbides by different laser parameters are shown in Supplementary Fig. 7, and the optimized conductivities can reach ~300 S cm^−1^. Such conductivity, although not compatible with metal, is at a range of graphite (perpendicular to basal plane), amorphous carbon, carbon nanotube forest, and highly doped semiconductor^41^. The carbides hold BET surface area from ~10–30 m^2^g^−1^ (Supplementary Fig. 8). The electrically conductive porous materials can perform as electrodes for many electrochemistry applications such supercapacitor and battery^42^. A few carbide materials (Mo_2_C, WC, and etc) show remarkable electrocatalysis activity such as hydrogen evolution reactions^32^. The laser direct printed carbide electrodes are promising to be implemented in Noble-metal free electrocatalytic nanodevices. As shown in Table 1 and Supplementary Fig. 9, the concentration of gelatin is essential for production of the carbide: above 10% all the products are in the form of carbide and carbon while below that concentration the product forms MoO_2_ and lower energy Mo_2_C. As revealed in Supplementary Fig. 10, the Mo-gelatin composite leads to the highest IR absorption at the typical wavelength of 10.6 μm compared to gelatin containing the same concentration of other metal ions^43,44^. W^6+^ and Co^2+^ gelatin induced carbides with metal phases, and both were metal/carbide composites represented by MC_x_ (M = W, Co, Supplementary Fig. 5). The Ti^4+^ and Zr^4+^ in the gelatin hydrogel are converted to composite materials of carbides and oxides while Ni^2+^ and Fe^3+^ are converted to metal oxides with small portions of carbide. Zn^2+^ does not produce any crystalline phase (Supplementary Fig. 11).

Two critical parameters determine which mechanism and product are observed when forming laser-induced ultrathin TMC. The first key parameter is the “effectiveness” of energy absorbed by the metal-gelatin template, which is determined by the absorption of the ion, polymer and metal-ligand interactions. An additional critical factor is the activation energy of carbonization. Using Mo^5+^ and Ti^4+^ as two examples, Mo-gelatin can be effectively converted to MoC_x_ at temperatures above 2000 K, while the ineffective energy conversion in the Ti-gelatin solution only activates the oxidation process to a more stable TiO_4_ phase. Even with the same component of metal gel and similar input laser power, a UV laser-induced metal and metal oxide phases instead of carbide phases. One possible reason is the lower temperature generated by lower absorption of UV power in metallo-hydrogel (Supplementary Fig. 12).

Further characterization of laser-induced MoC_x_ is shown in Supplementary Fig. 13–14. We scratched off the as synthesized carbide and used high power ultrasonic processing to disperse flakes in ethanol (~20 μm in lateral size), which are then drop-cast onto silicon oxide. Atomic force microscopy (AFM) showed a thicknesses of carbide flakes between 10–20 nm, mostly <15 nm (Supplementary Figure 14).

### Flexible energy storage devices with wide operating temperature range

**Fig. 2:**
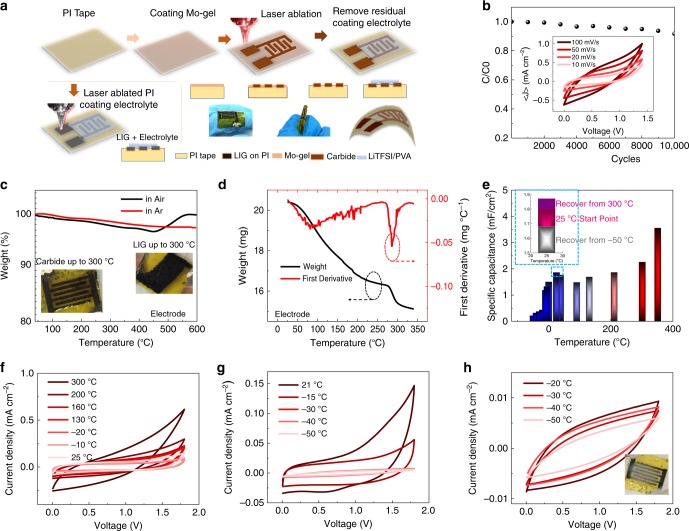
Flexible supercapacitor made of MoC_x_ with a wide operating temperature range. **a** Processing schematic of fabricating the flexible MoC_x_ supercapacitor. **b** Long-term retention of MoC_x_ supercapacitor. Inset: cyclic voltammetry (CV) at different scanning rate. **c** Thermogravimetric Analysis (TGA) of MoC_x_. Inset, optical images of Carbide and LIG electrodes heated up to 300 °C. The molybdenum carbide electrodes could withstand temperatures reaching 300 °C while the laser-ablated graphene on PI in the below image decomposed to a black color. **d** TGA of Li-rich PVA electrolyte. **e** Specific capacitance recovers during the temperature cycle, and high-temperature performance of the MoC_x_ electrodes. Inset: specific capacitance recovery in the temperature cycle. **f** CV results of carbide in LiTFSI/PVA/H_2_O electrolyte over the temperature cycle. **g**–**h** Low temperature performance of the MoC_x_ electrodes

The nanocrystalline porous ultrathin flakes provide exceptionally high surface area and exposed edges, which result in a high specific capacitance up to 55 F g^−1^ in 1 M LiCl and long-term stability of 10,000 cycles (Supplementary Fig. 15). Flexible, high performance MoC_x_ supercapacitor is patterned onto commercial Polyimide (PI) substrate. As shown in Fig. 2a, the Mo-gel is spin coated onto the PI tape, onto which interdigitated electrodes are then patterned. Unconverted Mo-gel is washed out, and highly concentrated PVA/LiTFSI electrolyte (20 molal) is coated. Supercapacitor device performance at different scanning rate is shown in Fig. 2b and Supplementary Fig. 16. The MoC_x_ supercapacitor shows over 90% maintenance of specific capacitance after 10,000 cycles (Fig. 2b). Lasing the commercial PI tape could induce porous graphene^33^, which will benefit the binding between laser-sculptured-carbide (LSC) and the flexible substrate both mechanically and electrically. Although LIG electrodes have also been used in supercapacitor (Fig. 2c), their temperature resilience is not compatible as laser scribed carbide^45^. As shown in in Fig. 2c, LIG electrodes in electrolyte turned to black color and damage in elevated temperature, while the LSC electrodes remains well functioning up to 300 °C. TGA of ultrathin-MoC_x_ exhibits high-temperature stability up to 450 °C in air (Fig. 2c), which outperforms the previously reported MXene thermal stability temperature of ~200 °C in air^17^.

As shown in Fig. 2d, the electrolyte employed is a high concentration of 20 m LiTFSI/PVA with thermal stability up to 300 °C. Based on the freezing point depression^46^, which is positively correlated to the ion concentration, freezing point would possibly decrease to −74 °C as estimated in Supporting Information. The LSC film achieved low electrical resistivity around 50 Ω sq^−1^ and the resulting micro supercapacitor prototype showed good specific capacitance (2mF cm^−2^) at 100 mV s^−1^ (Fig. 2e). This flexible supercapacitor showed stable operation under charge-discharge cycling over a wide range of temperatures from −50 °C up to 300 °C (Fig. 2e–f). The cyclic voltammetry (CV) measurements at 25 °C before and after the harsh environment tests at 300 °C clearly show no decrease in performance for the MoC_x_-based supercapacitors (Fig. 2e). At low temperatures down to −50 °C, the MoC_x_-supercapacitor maintains functionality, with no perceptible irreversible damage to the electrodes according to the recovery of the CV curves after defrosting (Fig. 2f–h). Below −30 °C a sharp decay in capacitance is observed due to the low ion mobility in the freezing electrolyte. Further modifications such as adding anti-freeze agents or changing the solvent could stabilize the electrolyte at such low temperatures.

### Solar-energy harvesting membrane made of carbide

**Fig. 3:**
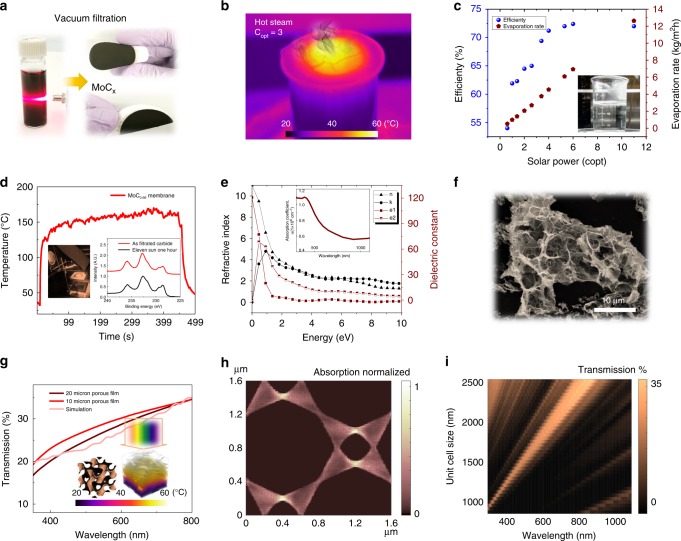
Laser-sculptured carbide for solar-steam generation membrane. **a** Fabrication schematic of carbide membrane. Laser-induced carbide is dispersed in DI water, which is condensed to a flexible membrane by vacuum filtration. **b** Solar-steam generation using carbide membrane to harvest solar-thermal energy. **c** Evaporation rate and energy efficiency of carbide membrane under different incident solar power. **d** Temperature file of MoC_1-x_ under extreme solar irradiation (11 sun, inset). XPS of MoC_1-x_ Mo_3d orbital before and after 11 sun exposure for 1 h. **e** Simulated refractive index, dielectric constant, and absorption coefficient of MoC_1-x_ by density functional theory. **f** SEM of laser structured porous MoC_x_ with connected curved surface. **g** Transmission spectrum of 10 μm and 20 μm porous carbide thin films. Inset: simulated transmission curve of approximate carbide structure (inset) and simulated temperature profile in carbide unit cell. Incident light power C_opt_ = 3 (3 kW m^−2^). **h** Simulated absorption of 500 nm incident light within a unit cell (1.6 μm) to illustrate the energy localization within the porous carbide structure. **i** Transmission intensity of different wavelengths in different approximate unit cell sizes

In contrast to chemically exfoliated MXenes and CVD synthesized ultrathin carbides, laser-sculptured carbide possesses a built-in interconnected curved surface, which could enable the tailored design of 3D morphologies consisting of interconnected ultrathin materials. One way to take advantage of such structures is in light capture; for example, the highly porous and curved carbide “walls” can efficiently harvest solar energy and transfer it to water for the generation of steam. Laser-sculptured carbide is sonicated in a water/ethanol mixture to detach from a glass substrate. Vacuum filtration yields a flexible membrane on versatile substrate support including Polytetrafluoroethylene (PTFE), Polyvinylidene (PVDF), and Polyethersulfone (PES) (Fig. 3a, Supplementary Fig. 17). The carbide membrane has strong absorption of a wide range solar spectrum, which induce local heating-driven evaporation at the water-air interface^47^. The solar-driven energy conversion at the interface reduce the energy input for bulk evaporation, which improve the steam productivity as shown in Fig. 3b–c^48^.

Under 3 sun illumination (3 kW m^−2^), enhanced solar-energy absorption generates a relatively higher temperature (60 °C) at the water-air interface compared to other photo-absorbing membranes such as carbonized wood (~40 °C)^49^, wood-CNT composite (~50 °C)^50^, graphite-carbon foam double layer structure (<40 °C)^48^ and etc. The evaporation rate and energy efficiency of solar-steam generation under different solar incident power is shown in Fig. 3c and Supplementary Fig. 18. Such energy efficiency is comparable to the membranes mentioned above, yet lower than a recently reported nano-hierarchical gel having an extremely high efficiency up to 90%^51^. No specific thermal management is deployed to decrease the thermal loss, which can be improved by adding an insulation layer^47,48^ to further increase the energy efficiency. Nevertheless, the high-temperature-resilience of the carbide enables steam generation in harsh environments that could burn or destroy many other polymer-based membranes^51^. The MoC_x_ reached ~160 °C within 60 s (Fig. 3d), under 11 sun exposure (Fig. 3d, inset). Near identical X-ray photoelectron spectroscopy (XPS) of MoC_x_ Mo_3d orbital before and after 11 sun exposure for one hour implies the carbide is stable in extreme solar radiation (Fig. 3d, inset), while a MoS_2_ membrane (Supplementary Fig. 19)^52^ is oxidized shown from its XPS Mo_3d orbital (Supplementary Fig. 20) in comparison. From the water transport analysis that we add to Supplementary Fig. 20 and Supplementary Table 2, dry-out is not happening at the membrane-water interface. Capillary force can drive water to the membrane surface. Carbide membrane shows a permeability of 1973.4 LMH bar^−1^, which falls to the range of membrane with pore size from hundreds of nanometers to micrometers^53^.

We employ density function theory (DFT) and finite differential time domain (FDTD) simulation to understand the solar-energy localization within carbide porous structure. Due to the lack of available reference data for experimentally measured optical properties of MoC_1-x_, we employ first principles simulations of the carbide dielectric constant and refractive index (Fig. 3e). The FDTD model is based on the SEM image in Fig. 3f, and a “gyroid-like” curved structure with variable unit cell is used to represent the porous carbide (Fig. 3g inset). The measured optical transmission for both 10 μm and 20 μm thick porous carbide membranes are shown in Fig. 3g, in good agreement with the computed optical transmission for the same wavelength range. All details of the DFT simulation and FDTD simulation are in the supporting information (Supplementary Fig. 21). Figure 3h is one example of optical absorption (500 nm incident light) projected onto the x–y plane in a unit cell (1.6 × 1.6 μm). Figure 3i shows the absorption intensity trend of different wavelength in different size carbide cells.

## Discussion

In conclusion, we have developed a versatile approach for the synthesis of microporous ultrathin polycrystalline carbides (~10 nm crystallinity) with ultrathin “wall” structures (~10–20 nm), using lamellar templated metal ion (Mo, W, and Co) containing gelatin (metallo-hydrogel) and processing with a low-cost and fast CO_2_ laser cutter in ambient environment. The IR energy generates local high temperatures which induce a rapid carbon uptake to form carbon-deficient phases (MoC_x_, WC_x_, and CoC_x_), and sculptured interconnected curved structures during the sub millisecond “explosive” carbonization process. The laser-induced MoC_x_, as a conductive and refractory metallic material with hierarchical porosity (from microscale to nanoscale), was shown to be an appealing candidate for energy storage in lithium-based electrolyte (55 F g^−1^, discharged at 1 mV s^−1^). Direct patterned MoC_x_ supercapacitor on PI tape shows a specific capacitance up to 20 mF cm^−2^ at a discharge rate of 1 mV s^−1^, and 2 mF cm^−2^ at 100 mV s^−1^. This work provides a general approach for a low cost, room temperature, high yield and exceptionally fast synthesis of transition metal carbides (Supplementary Table 3), which also enable large-scale devices and potentially roll-to-roll manufacturing. The non-decorated surface with less functional groups (–OH, –COOH) provide higher temperature resilience than surface-function-rich and chemical resilience (Table 2). The MoC_x_ interdigitated electrodes used in conjunction with a Li-rich electrolyte (for lower freezing point) was demonstrated to function over a temperature range of −50 °C to 300 °C with no significant degradation while the laser-induced graphene decay in the same electrolyte. Laser sculpturing also provide hierarchical structure in multiple scale (ultrathin 2D like to 3D, Table 2), which also potentially bring intriguing properties and tunability in light and phonon transport. A post vacuum filtered MoC_x_ membrane, exhibited effective solar-steam generation due to the size variable “gyroid-like” curved surface, which enhanced solar-energy harvesting. The direct write patterning and sculpturing process opens these materials up to a broader window of applications in flexible electronics, energy storage, energy harvesting, and water treatment applications.

**Table 2:** Summary and comparison among laser-induced graphene (LIG), MXenes, and laser-sculptured carbide (LSC)

Materials	LIG	MXene	LSC (this work)
Thermal stability (TGA in air)	Start burning over 200 °C^45^	~200 °C^17^	450 °C
Resilience (in electrolyte)	<300 °C	NA	>300 °C
Dimension	2D->3D porous	Layered 2D	Ultrathin 2D like ->3D porous

## Methods

### Preparation of transition metallo-hydrogel

MoCl_5_, CoSO_4_, NiSO_4_, FeCl_3_, Zr(NO_3_)_4_, and Zn(NO_3_)_2_ (used as received from Sigma–Aldrich) were dissolved in deionized water, and WCl_6_ was dissolved in N-Methyl-2-pyrrolidone (NMP, Sigma–Aldrich) with a concentration of 2 m (m is mass molality). The hydrogel was made by dissolving either gelatin, PVP or PEO with 60% wt concentration. Tetrabutyl titanate were mixed with NMP with a concentration of 2 m, which showed much lower solubility of gelatin (<5 wt%). Such Ti-NMP-gelatin mixture could only make suspension rather than uniform solution.

### Synthesis of metal carbide by laser ablation

Metallo-gel precursor was spin coated onto glass and cured at 80 °C for 1 h to make a thin film, which was subsequently ablated by laser (IR 2 W; UV 1 W due to the limited of USB powered of UV laser. A commercial Universal VSL 2.30 was utilized to perform the laser ablation, with a CO_2_ laser tube and all built-in optics. Laser spot is focused on the top surface of spin-coated gel film, and the height of the sample is controlled by tuning the z-position of the supporting cutting table. Designed pattern is imported to a vector drawing software and engraved by communicating with the laser cutter as a printer. Unablated gel can be easily removed by rinsing the whole device in DI water for 1 min and air dry before test.

### Materials characterization

Scanning electron microscopy (SEM, FEI Quanta 3D), Transmission electron microscopy (TEM, FEI Tecnai) were employed to study the morphology and structure. X-ray diffraction (XRD, Bruker D8) is performed to study the crystallinity, and X-ray photoelectron spectroscopy (CHI) is used to study the surface element component of samples. Co source is used in the powder diffraction X-ray test.

### Electrochemistry test

Ag/AgCl was used as the reference electrode to study majorly carbide performance in different electrolytes. Magnesium acetate solution (~1 M, volumetric concentration), was purchased from BioUltra. NaCl (≥99%wt, Sigma–Aldrich) and LiCl (≥99.99% wt, Sigma–Aldrich) were dissolved in distilled water to make solution with concentration of 1 M (volumetric molarity). Concentrated sulfuric acid is diluted by deionized water to a volumetric molarity of 1 M. Linear sweep voltammetry, cyclic voltammetry, chronopotentiometry, and EIS impedance tests were performed by an electrochemistry workstation (Gamry Ref 600) with different modules. Powdery MoC_x_ flakes are grinded with PAN (1 wt%) dissolved in N-Methyl-2-pyrrolidone (NMP, Sigma–Aldrich) as binder, which is casted onto hydrophobic carbon paper (Toray Carbon Paper 060, Wet Proofed).

### Fabrication of supercapacitor of carbides on PI tape

Mo-gelatin (2 m MoCl_5_, 60 wt%) hydrogel is spin coated onto Polyimide tapes to form a thin film, which is dried in the oven at 80 °C for 30 min. Gel-on-PI film is ablated by IR laser with 2 W power at a speed of 200 mm s^−1^. Interdigit structure is patterned with the geometry design using CAD interface. Li-rich electrolyte (21 m LiTFSI) is dissolved in DI water with 10% PVA, which is deposited onto the interdigit electrodes to assemble a functional supercapacitor. WC_x_ and CoC_x_ supercapacitor are fabricated via the same process using W-gelatin (2 m WCl_6_, 60 wt%) and Co-gelatin hydrogel (2 m CoCl_2_, 60 wt%).

### Fabrication of ultrathin-TMC membrane

Laser-sculptured MoC_x_ was scratched off subtracted, dispersed in DI water and ultrasonic for 2 h at 80 °C. The supernatant was vacuum filtered using a porous Polytetrafluoroethylene (PTFE, 47 mm diameter, 0.45 μm pore size, Fisher Scientific, Fair Lawn, NJ, USA) and dried in the air for 24 h. Same process is performed to vacuum filtrate carbide membrane onto Polyvinylidene (PVDF, 0.45 μm pore size, 47 mm diameter, Sterlitech Corp) and Polyethersulfone (PES, 0.45 μm pore size, 47 mm diameter, Sterlitech Corp) membrane filters.

### Test of solar-thermal energy harvesting and steam generation

The experimental setup is equipped with a solar simulator (Newport 94023 A), and a balance, with data collection system (OHAUS, 420 g, with USB cable). The 47 mm membrane was exposed to solar illumination on the top of a baker (2 inch diameter) filled with DI water. IR camera was employed to record the temperature, with the balance to record mass loss.

### Density functional theory simulation details for MoC_x_ optical properties

All ab initio calculations were performed using the Vienna Ab Initio Simulation Package (version 5.4.4.18Apr17^54^). To obtain the dielectric tensor, the structure of MoC_1-x_ was first relaxed to ensure all the forces on the ions were smaller than 0.01 eV Å^−1^. Then dielectric tensor was calculated using the independent particle approximation, and subsequently the local field effects were included using the random phase approximation. All calculations were performed using Perdew–Burke–Ernzerh (PBE^55^) version of generalized gradient approximation with projected-augmented wave potentials^56,57^ and a wave function energy cutoff of 400 eV; the Brillouin zone was sampled with, respectively, 1728 and 4096, k-points, to confirm convergence. In calculating the frequency-dependent dielectric tensor, 2000 frequency grid points were used, and the actual number of bands (1280) was carefully chosen such that a considerable number of empty bands was included in the calculation.

## Supplementary information

Supplementary Information

## Data Availability

The data that support the findings of this study are available from the corresponding author upon reasonable request.
