# Supplementary material for: Laser-sculptured ultrathin transition metal carbide layers for energy storage and energy harvesting applications
Source: Nat Commun. 2019 Jul 15;10:3112. doi: 10.1038/s41467-019-10999-z (PMC6629648; doi:10.1038/s41467-019-10999-z)
Supplement: Supplementary file 1 — Supplementary Information [file 41467_2019_10999_MOESM1_ESM.pdf]

Supplementary Information

Laser-sculptured Ultrathin Transition Metal Carbide Layers for Energy Storage and Energy Harvesting Applications

Xining Zang,[1,2] Cuiying Jian,[1] Taishan Zhu,[1] Zheng Fan,[3] Wanlin Wang,[4] Minsong Wei,[2] Buxuan Li,[2] Mateo Follmar Diaz,[5] Paul Ashby,[6] Zhengmao Lu,[1] Yao Chu,[2] Zizhao Wang,[7] Xinrui Ding,[2] Yingxi Xie,[2] Juhong Chen,[2] J. Nathan Hohman,[6] Mohan Sanghadasa,[8] Jeffrey C. Grossman,[1*] & Liwei Lin[2*]*

1. Department of Materials Science and Engineering, Massachusetts Institute of Technology, Cambridge MA, USA, 02139
2. Mechanical Engineering & Berkeley Sensor and Actuator Center, University of California Berkley, Berkeley, CA, USA, 94704
3. Department of Engineering Technology, University of Houston, Houston, TX 77204, USA
4. College of Electronic Science and Technology, Shenzhen University, Shenzhen, China, 518060
5. Micro and Nanosystems, D-MAVT, ETHZ, Zürich, Switzerland, CH – 8092
6. Molecular Foundry, Lawrence Berkeley National Lab, Berkeley, CA, USA, 94720
7. School of Engineering and Applied Sciences, Harvard University, Cambridge MA, USA, 02138
8. Aviation and Missile Center, U.S. Army Combat Capabilities Development Command, Redstone Arsenal, AL 35898

* Corresponding author, email: xzang@mit.edu jcg@mit.edu lwlin@berkeley.edu

Supplementary information list:

Supplementary Figure 1. Spin coating fitted curve.
Supplementary Figure 2. X-ray diffraction from laser ablated molybdenum carbide.
Supplementary Table 1. Materials properties set up for temperature simulation.
Supplementary Figure 3. Temperature simulation and corresponding high energy phase in Mo-C diagram.
Supplementary Figure 4. Nanostructure of laser ablated MoC_x.
Supplementary Figure 5. Laser induced carbide from tungsten and cobalt ion dissolved in gelatin media.
Supplementary Figure 6. Laser ablation product at different power.
Supplementary Figure 7. Conductively of MoC_x ablated with different laser power and scanning speed.
Supplementary Figure 8. Isothermal absorption curve of laser ablated carbide.
Supplementary Figure 9. Different product derived from Mo-gel with different gelatin concentration.
Supplementary Figure 10. IR absorption of hydrogel made of 60% gelatin with different metal ions at 2m.
Supplementary Figure 11. Laser converted gelatin hydrogel with other metal ions.
Supplementary Figure 12. Laser induced metal/metal oxide film by UV laser.
Supplementary Figure 13. X-ray photoelectron spectroscopy (XPS) characterization of intrinsic properties of laser ablated 2D-MoC_x.
Supplementary Figure 14. Atomic force microscopy (AFM) mapping of 2D-MoC_x flakes.
Supplementary Figure 15. Electrochemical energy storage property of 2D-MoC_x.
Supplementary Figure 16. Electrochemical performance of MoC_x, WC_x, CoC_x interdigit supercapacitor, using LiTFSI-PVA electrolyte.
Supplementary Figure 17. MoC_x membrane filtrated onto different substrates including PTFE, PVDF and PES.
Supplementary Figure 18. Solar steam generation energy efficiency of MoC_x membrane under eleven sun incident power.
Supplementary Figure 19. Solar heating profile and thermal stability of a MoS_2 membrane, made by the method in a published paper for comparison.
Supplementary Figure 20. Water transport analysis, with a simplified model of water through a cylindric pore.
Supplementary Table 2. Measured permeability of MoC_x on PTFE substrate.
Supplementary Figure 21. FDTD simulation results of optical absorption within the porous carbide structure.
Supplementary Table 3. Summary of State-of-Art Manufacturing Processes for Metal Carbides
Supplementary Note 1-7

$$t = a\omega^{-b}$$

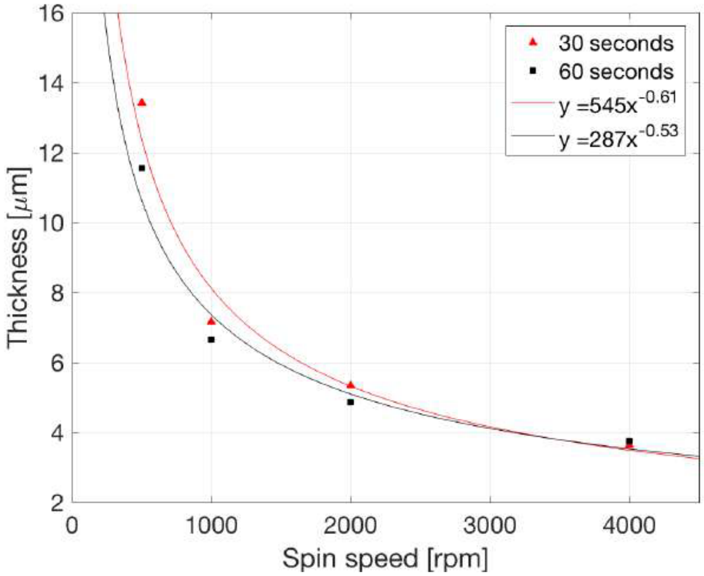

Supplementary Figure 1. Spin coated Mo-gel thickness and fitting curve.

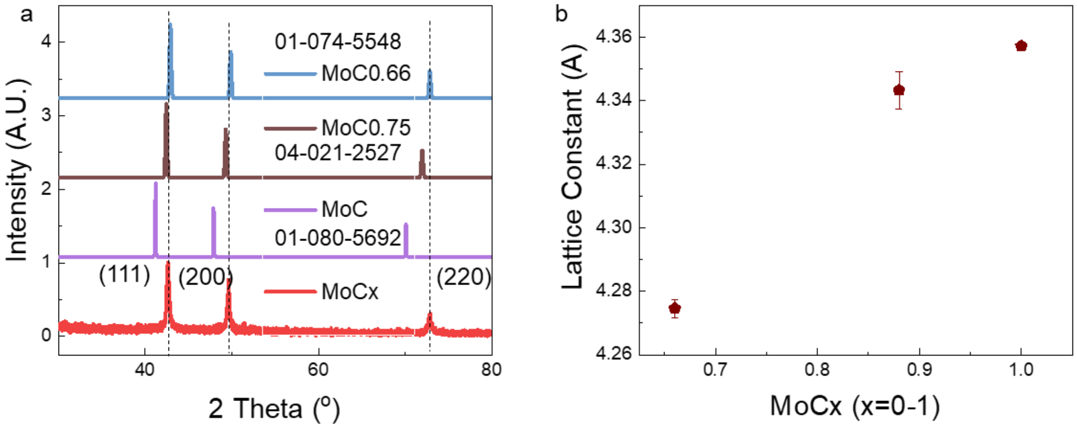

Supplementary Figure 2. X-ray diffraction from laser ablated molybdenum carbide comparing to MoC, $MoC_{0.75}$[1], and $MoC_{0.66}$ from ICSD database. Density function theory simulation of lattice constant of a unit cell of α-MoC_x (x=0.66,0.88,1).

Supplementary Table 1. Materials properties set up for temperature simulation.

	ρ Density (Kg m^{-3})	k Thermal conductivity (W m^{-1}K^{-1})	C_p Specific Heat (J Kg^{-1}K^{-1})	Absorption at 10.6μm[2] (a.u.)	P_laser Simulation Heat Power (W) (set 4W)
$MoCl_5$+Gelatin	720	0.375-0.61[3]	1600[4]	0.35	1.4
$MoCl_5$+PVP	1144	0.27[5]	3963[6]	0.32	1.28
$MoCl_5$+PEO	1210	0.15	2974-3410[7]	0.31	1.24

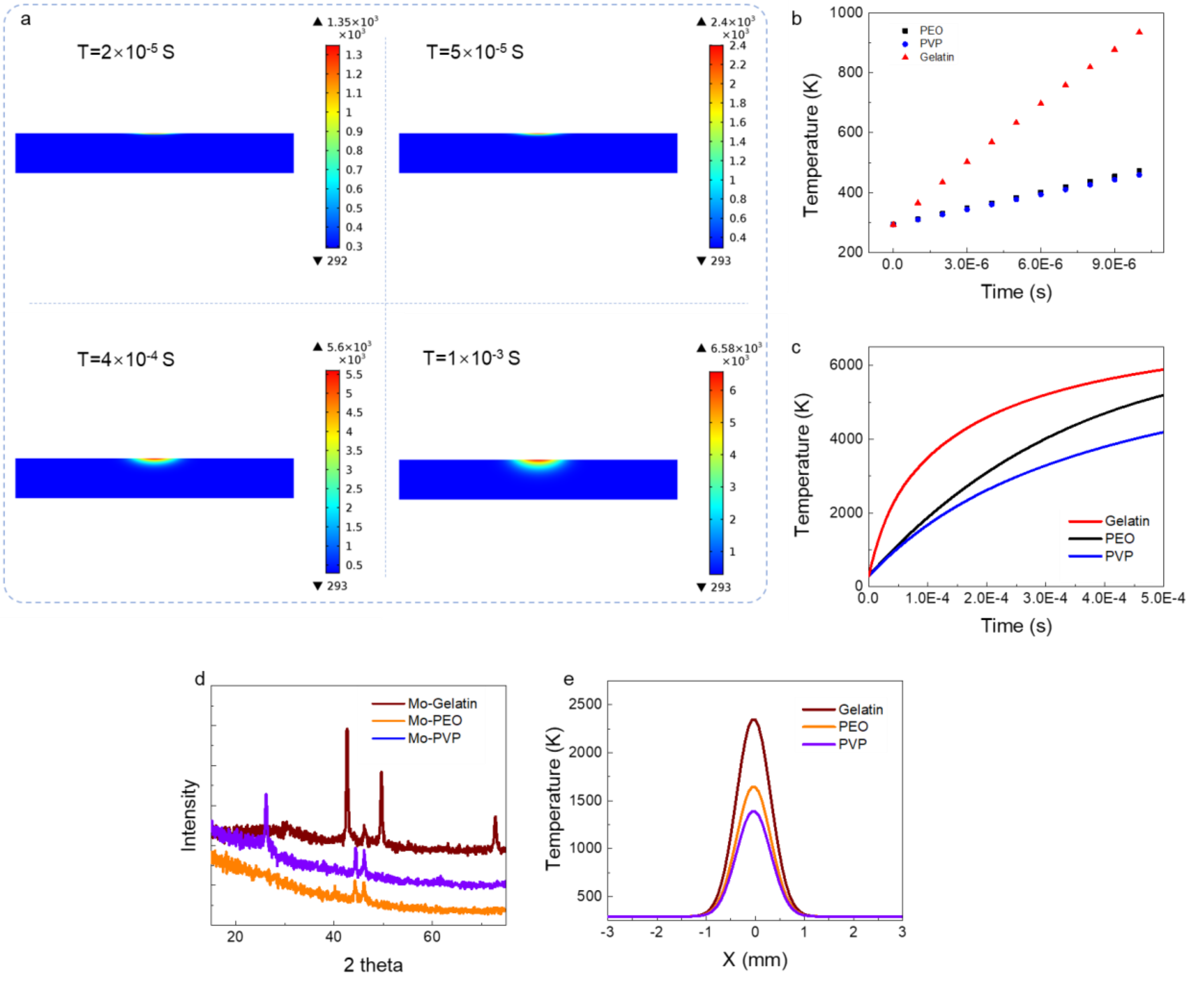

Supplementary Figure 3. Temperature simulation and the corresponding high energy phase in Mo-C diagram. (a) Cross-section view of Mo-gelatin sample. Illustrating the vertical temperature profile with different time scale. (b) Temperature versus time (time interval: 1E-5s, 0 to 5E-4s). (c) Temperature versus time (time interval: 1E-6s, 0 to 5E-5s). (d) X-ray diffraction (XRD) of laser ablated product of hydrogel made of 2m Mo^{5+} in different polymer media. (e) Simulated temperature of Mo-Gelatin assuming the activation time constant is 5×10^{-5}s, showing a Gaussian distribution of temperature up to 2500 K. Temperature profile of Mo-gelatin, Mo-PVP and PEO-PVP ablated by 2W IR laser, with a focus radius of 100 μm (0.2 mm diameter).

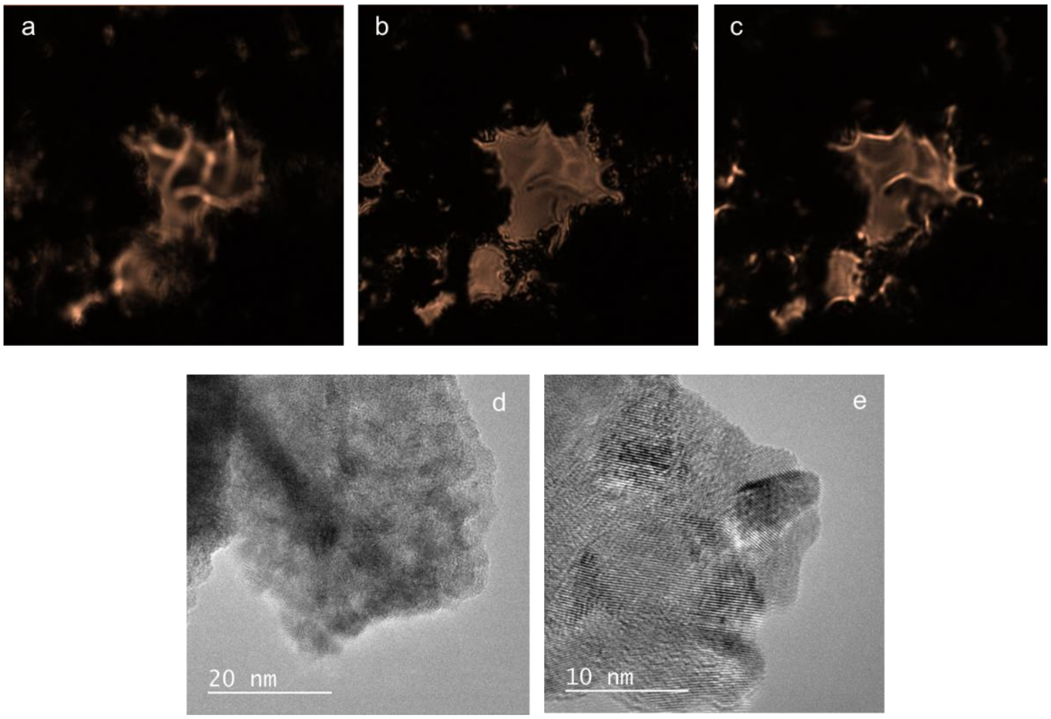

Supplementary Figure 4. Nanostructure of laser ablated MoC_x. (a-c) Three slices of the 3D tomography of a selected area of laser ablated MoC_x sample with a projected size of 80μm×80μm. (d-e) TEM images of laser ablated MoC_x.

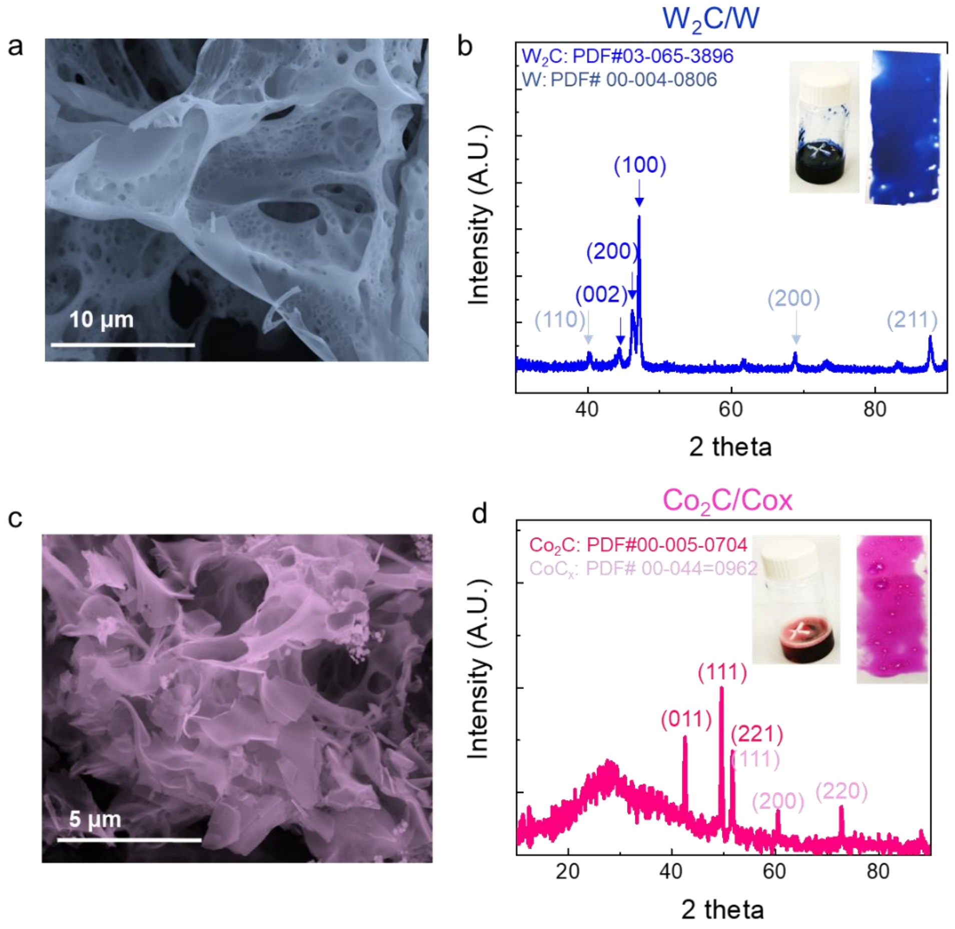

Supplementary Figure 5. Laser induced carbide from tungsten and cobalt ion dissolved in gelatin media. (a) SEM image of laser induced WC_x from W-gel. (b) XRD of laser induced WC_x, insets are optical images of the W-gel and casted thin film on glass. (c) SEM image of laser induced CoC_x from Co-gel. (d) XRD of laser induced CoC_x, insets are optical image of the Co-gel casted thin film on glass.

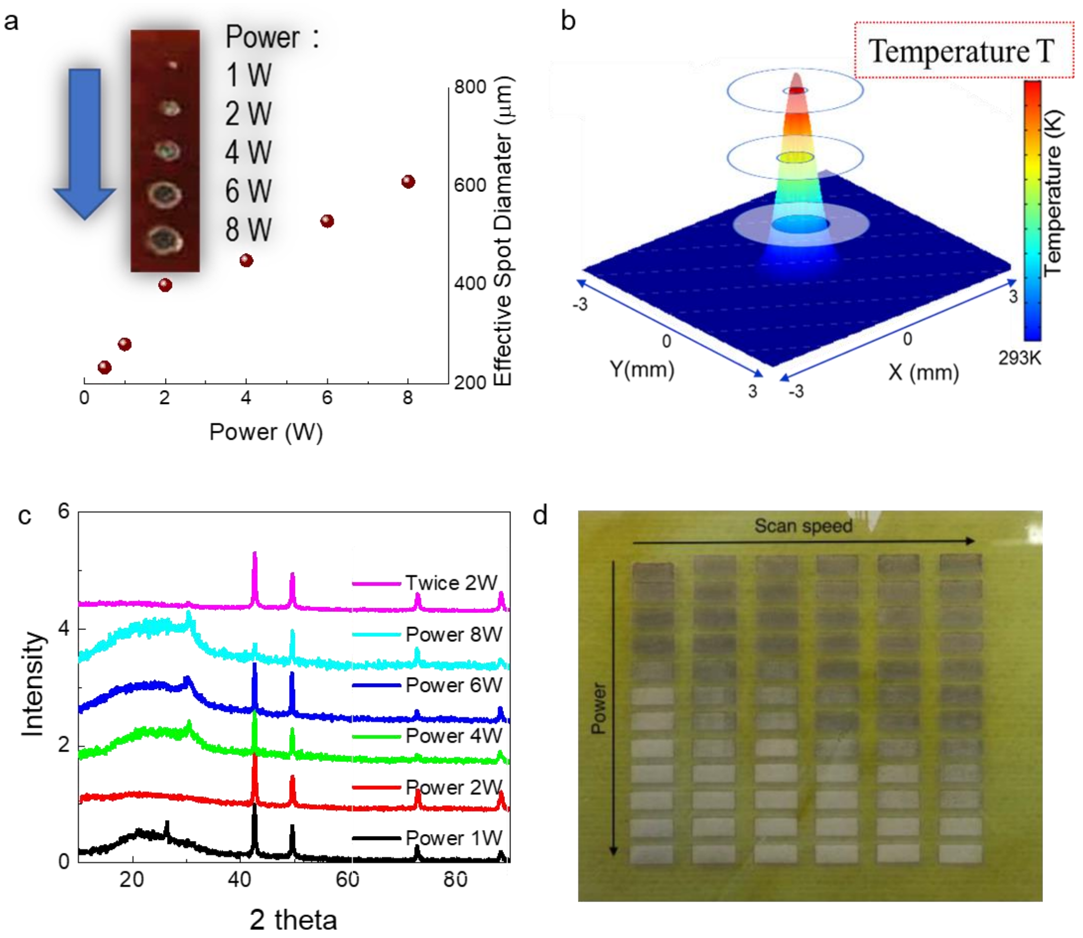

Supplementary Figure 6. Laser ablation products under different power levels. (a) The diameter of laser spot increases with input laser power. (b) Temperature distribution of the laser ablation result. The threshold temperature determines the cut-off size of the laser spot. (c) XRD of laser ablated carbide by different power. (d) Optical image of laser-induced carbide with different power and scan speed. The cut-off area with temperature higher than the threshold value for the conversion of carbide is determined by the optical image of the pulse spot size in **Supplementary Figure 6a,b**. It is found that the laser ablation process of molybdenum carbide shows a high-power tolerance at a wide range (from 1W to 8W). Higher laser power results in larger ablation spots while the carbide product after the conversion process is the same (**Supplementary Figure 6c**).

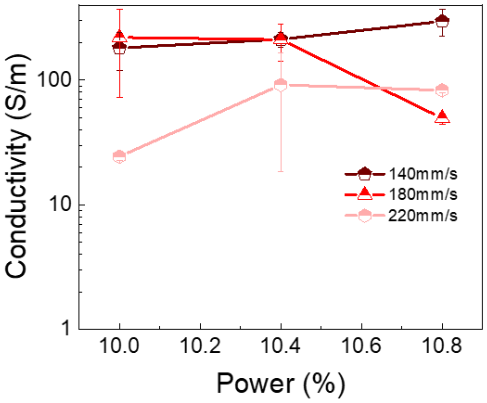

Supplementary Figure 7. Conductively of MoC_x ablated with different laser power and scanning speed.

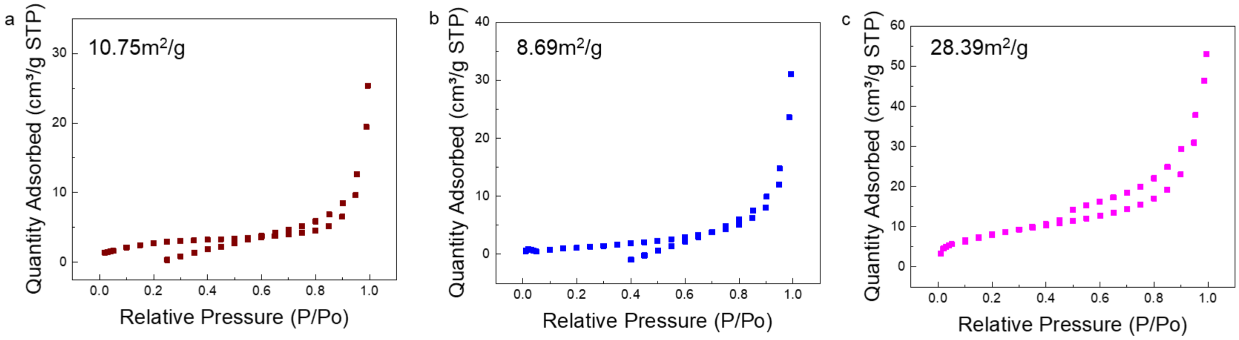

Supplementary Figure 8. Isothermal absorption curve of laser ablated carbide: (a) MoC_x, (b) WC_x, and (c) CoC_x.

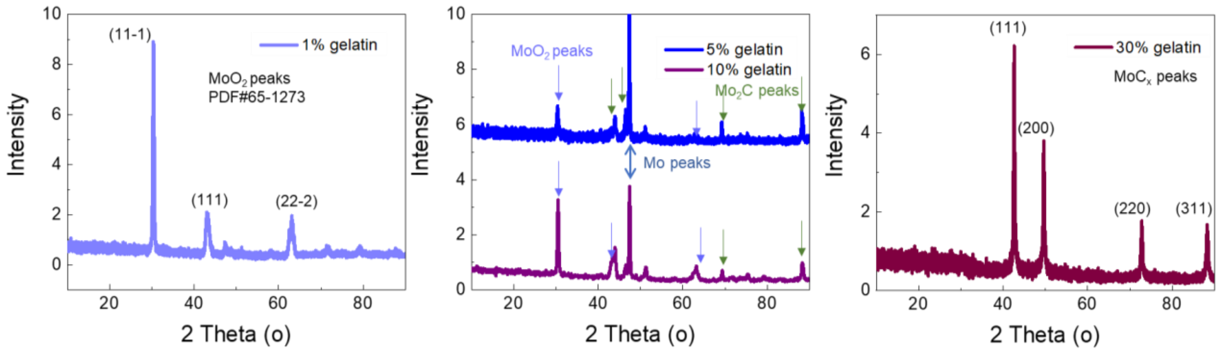

Supplementary Figure 9. Different products from the laser ablation process with different gelatin concentrations. The salting out effect of Mo in between the gelatin template layers require gelatin "ligands", and the absorption of IR energy by gelatin is essential in the conversion process. Samples with low gelatin concentrations will result in low local temperatures to produce MoO_2. MoO_2 PDF# 65-1273.

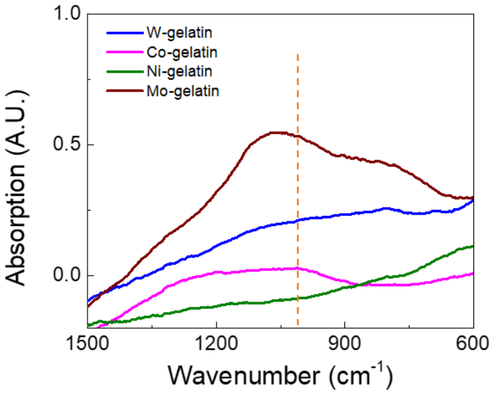

Supplementary Figure 10. IR absorption of hydrogel made of 60% gelatin with different metal ions at 2m.

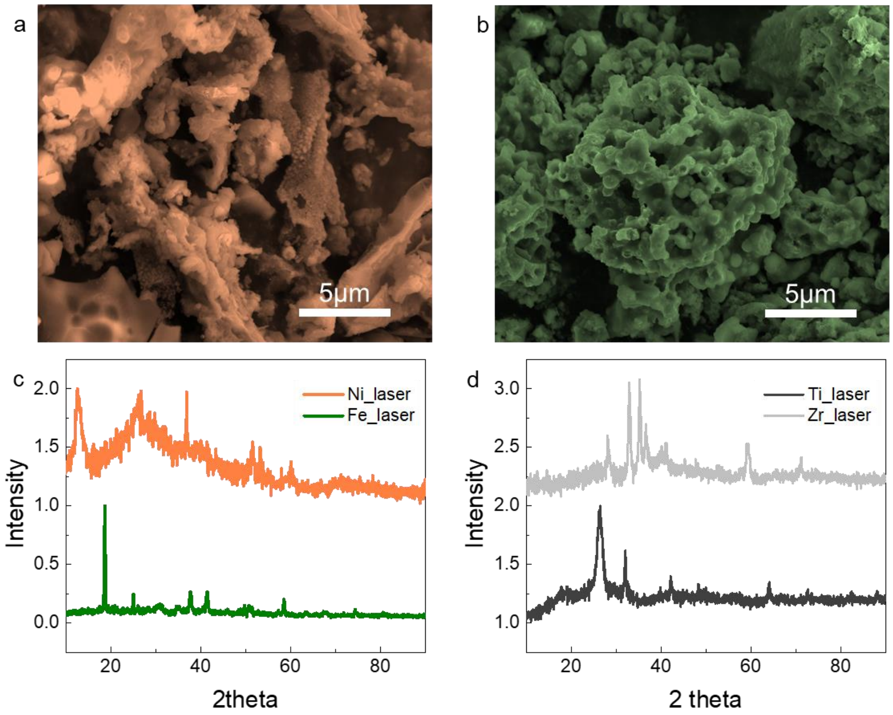

Supplementary Figure 11. Laser converted gelatin hydrogel with other metal ions. (a) and (b) SEM images of particles and chunk structure of laser ablated iron-gelatin and nickel-gelatin template. (c) XRD of samples in (a) and (b). (d) XRD of oxide/carbide hybrid by Ti^{4+} and Zr^{4+} precursors.

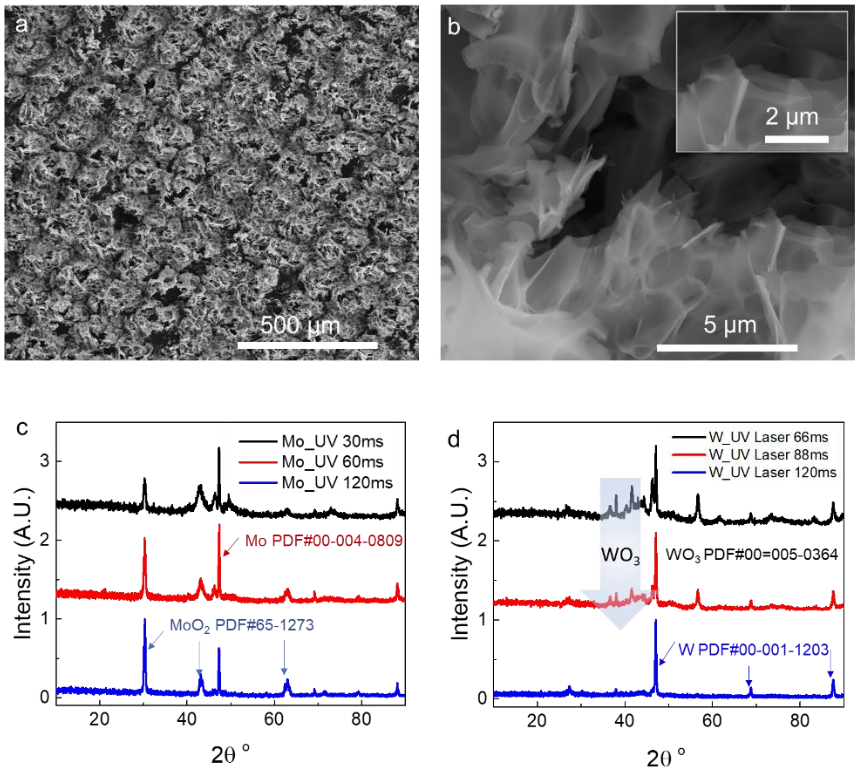

Supplementary Figure 12. Laser induced metal/metal oxide film by a UV laser (2W, 500nm). (a)(b) SEM image of UV laser ablated Mo-gelatin template. The inter-pulse distance in UV laser is much higher than that in the IR laser, and thus induced separated "sculpture" rather than continuous film. (c) XRD of Mo-gelatin with different burning time, showing consist product of Mo/MoO_2 composite. (d) XRD of W-gelatin with different burning time, showing the product of W/WO_x with shorter burning time. With a burning time of 120ms, the product is highly pure W.

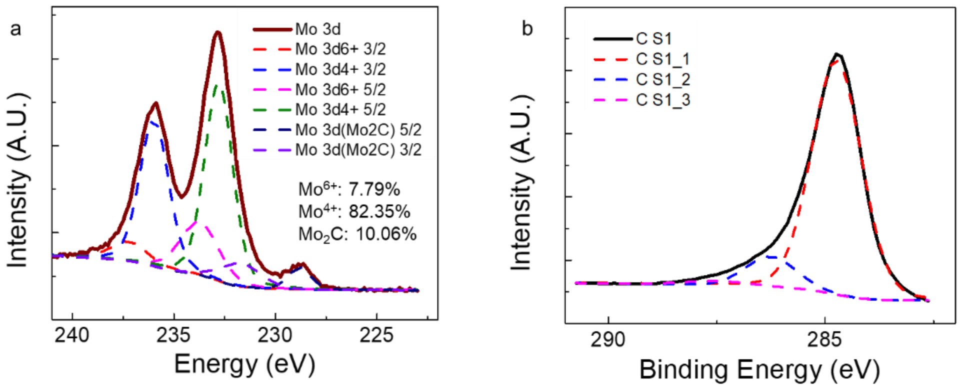

Supplementary Figure 13. X-ray photoelectron spectroscopy (XPS) characterization of the intrinsic properties of laser ablated MoC_x. (a) XPS spectrum of Mo_3d orbital of the laser induced MoC_x sheets. Quantitative summary is added in the figure. (b) C-1s orbital of the laser induced MoC_x sheets. Splitting of Mo_3d orbital could be due to the surface oxidation during the laser ablation in ambient environment, while small portion of split C_1s orbital could be attributed to the absorbed carbon oxide species on TMC surface.

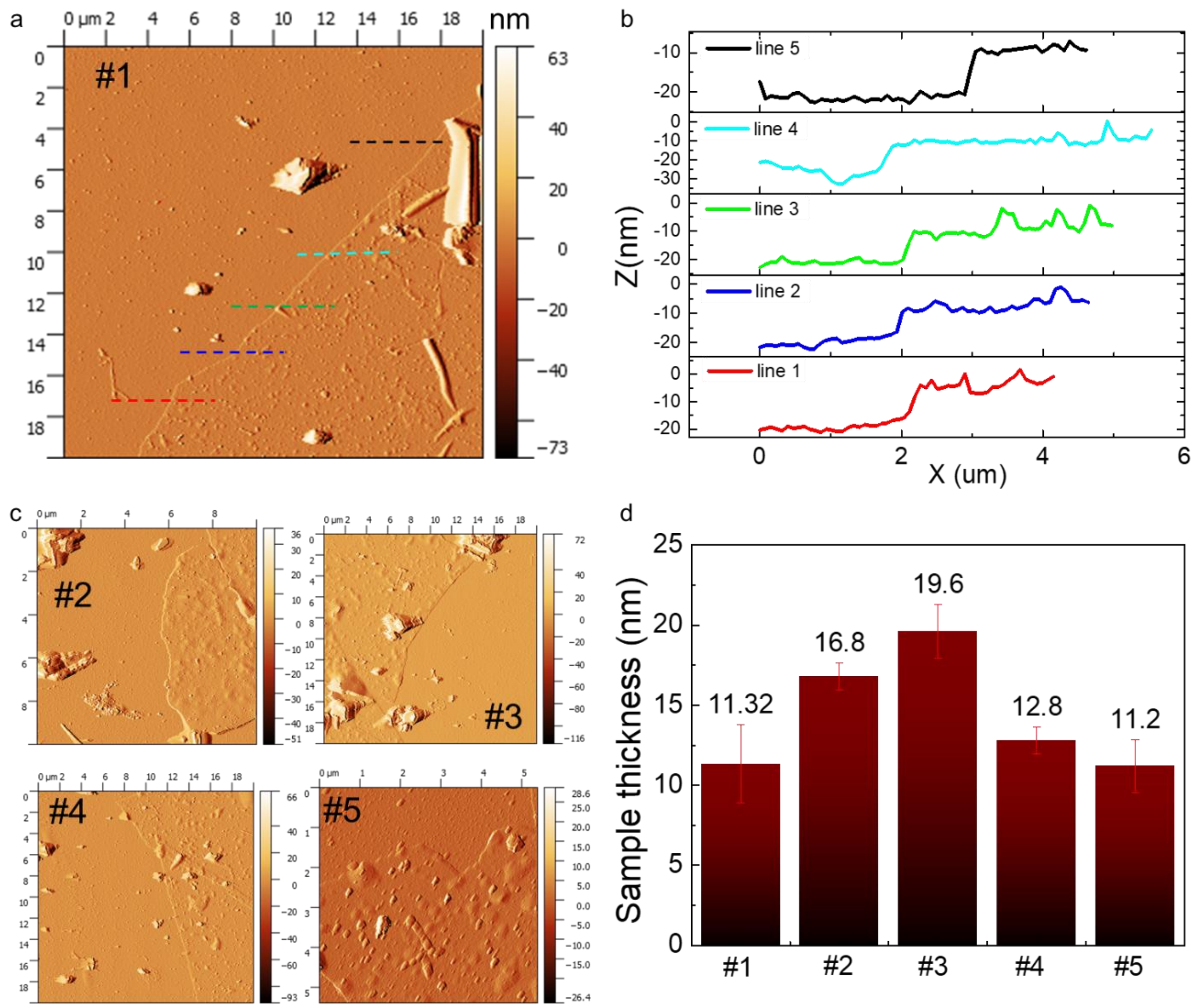

Supplementary Figure 14. Atomic force microscopy (AFM) mapping of MoC_x flakes. (a, b) Atomic force microscopy (AFM) mapping on a sheet (#1 sample) of MoC_x, showing thickness down to ~11.3 nm. (c) AFM mapping of sample #2~#4. (d) Statistical results of MoC_x sample #1~#5.

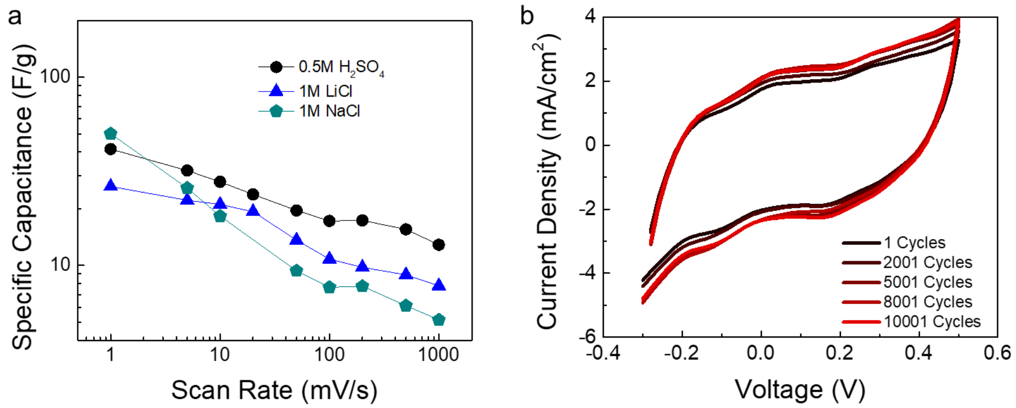

Supplementary Figure 15. Electrochemical energy storage property of MoC_x. (a) Specific capacitances results of MoC_x electrode in sodium chloride, sulfuric acid, and lithium chloride, respectively. (b) Long term retention of MoC_x electrodes in 1M LiCl for 10,000 cycles.

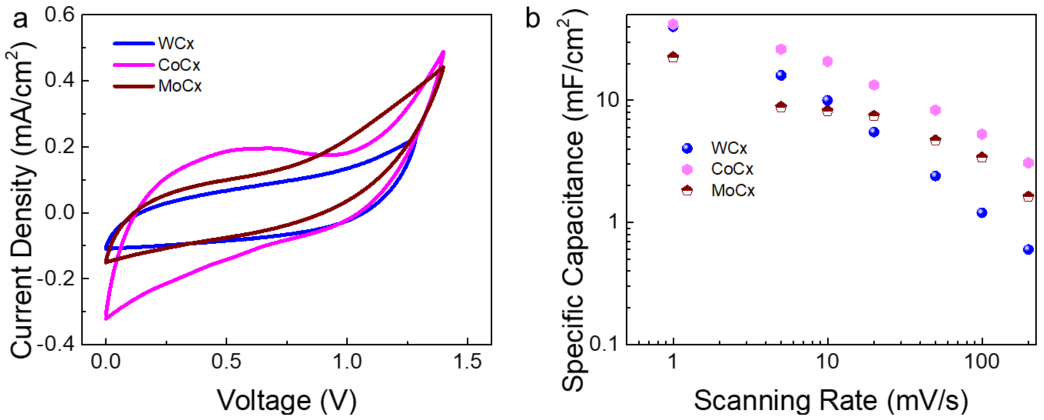

Supplementary Figure 16. Electrochemical performance of MoC_x, WC_x, CoC_x interdigit supercapacitor, using LiTFSI-PVA electrolyte. (a) CV results of different carbides, at a scan rate of 20 mV s^{-1}. (b) Areal specific capacitance of MoC_x, WC_x, and CoC_x supercapacitors at different scanning rate. Supercapacitor devices are presented in **Supplementary Figure 16** and discussed supporting information. WC_x holds smaller specific capacitance at higher scanning rate, which is related to its smaller surface area. CoC_x shows slightly higher specific capacitance, while the obvious redox peak near 0.5V might introduce oxidation in the nonpure CoC_x carbide phase.

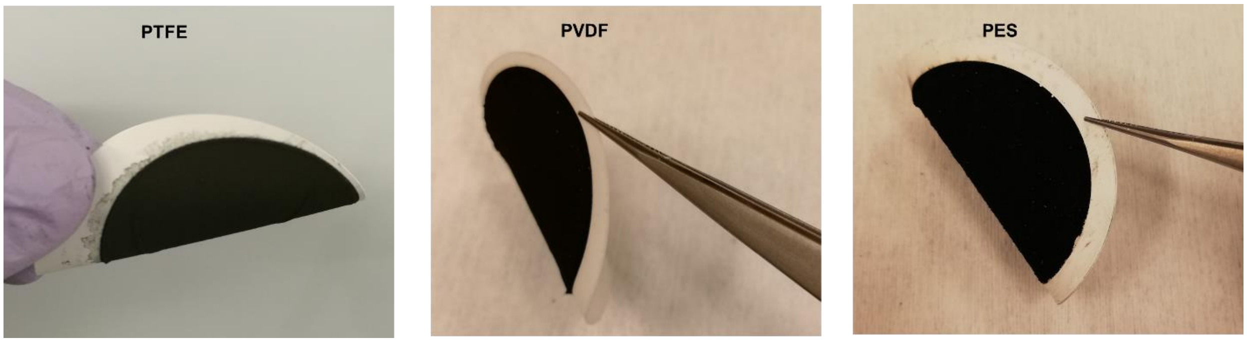

Supplementary Figure 17. MoC_x membrane filtrated onto different substrates including Polytetrafluoroethylene (PTFE), Polyvinylidene (PVDF) and Polyethersulfone (PES).

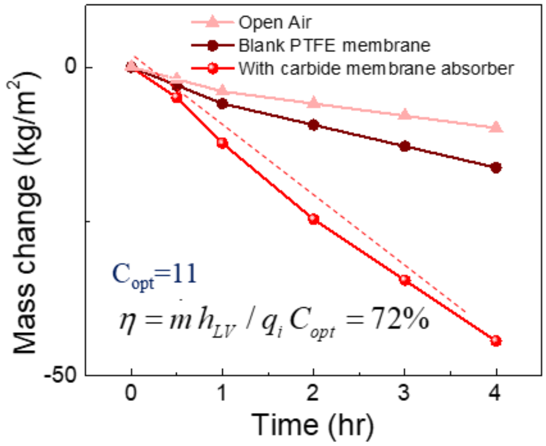

Supplementary Figure 18. Solar steam generation energy efficiency of MoC_x membrane under eleven sun incident power.

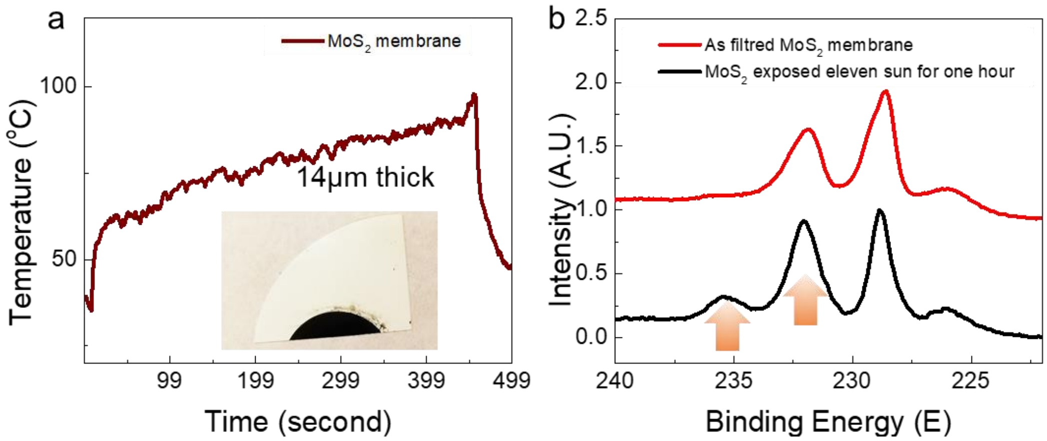

Supplementary Figure 19. Solar heating profile and thermal stability of a MoS_2 membrane, made by the method in a published paper for comparison [8]. (a) The temperature profile of the 14μm thick MoS_2 membrane under 10 sun, which reach below 100 °C with in 8min. (b) XPS result of Mo 3d orbital before and after 10 sun radiation for one hour. The binding energy shift, and the Mo^{6+} 3d_3/2 peak pop up after radiation indicating the oxidation of the MoS_2 membrane.

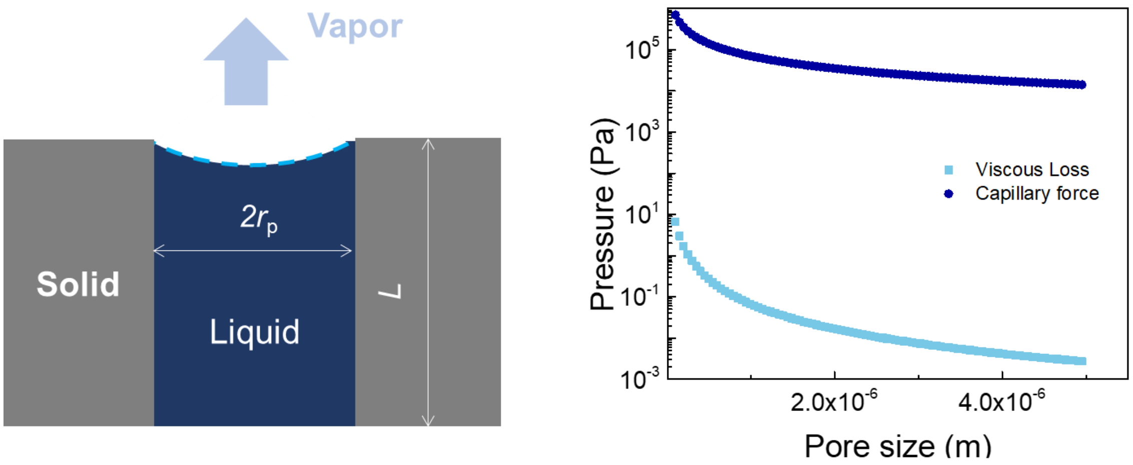

Supplementary Figure 20. Water transport analysis, with a simplified model of water through a cylindric pore. Assume the membrane is 10μm thick, solar power is one sun (1kw m^{-2}), energy efficiency 55%.

Supplementary Table 2. Measured permeability of MoC_x on PTFE substrate.

**Δ mass**	**Δ time**	**Pressure**	**Area**	**Permeability**
L	hour	bar	m2	LMH bar^{-1}
0.00528	0.08333333	0.68947448	4.6566E-05	1973.44778

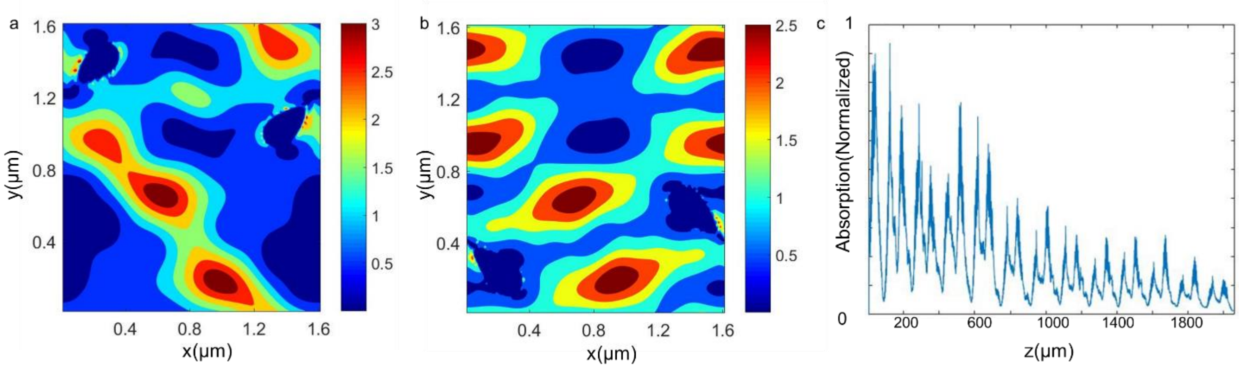

Supplementary Figure 21. FDTD simulation of visible light absorption in the porous carbide structure. (a)&(b) cross-section view of electric field with in the porous carbide structure at selected z-positions in the unit cell. Incident electric field is normalized to 1, while the porous curved surface enhances the electric field over 1 which enhance the absorption. (c) Light absorption with in the porous structure at different z-height, light input at z=0. A plane-wave source with a wavelength of 500 nm was applied to illuminate perpendicular to the gyroid structure from the (100) direction. The boundary condition in the light incidence direction was set to provide a perfectly matched layer, and in the horizontal direction the boundary condition was set to the periodic boundary condition. Simulations in which the incident light showed both *s* polarization and *p* polarization were conducted.

Supplementary Table 3. Summary of State-of-Art Manufacturing Processes for Metal Carbides

	Manufacturing Process & Materials	Temp. (℃)	Time (h)	Key Products	Ref.
**Molybdenum Carbides (not ultrathin 2D materials)**	Reduction: MoO_2 in CH_4–H_2	675~1100	10~40	α-MoC_{1-x}, β-Mo_2C	[9, 10, 11]
	CVD: Cu/Mo foil with CH_4–H_2; $MoCl_5$, benzene	800~1100	1	α-MoC_{1-x}, β-Mo_2C	[12, 13, 14]
	Pyrolysis: $Mo_3O_{10}(C_6H_8N)_2 \cdot 2H_2O$, $Mo(CO)_6$, $Cp_2Mo_2(CO)_4$	600~1000	2~5	β-Mo_2C	[15, 16]
	Ball-milling & Calcination: MoO_3 + carbon source (CNT or graphene)	1200	24	Mo_2C	[17, 18]
	Hydrothermal: F127-PMA gel or $(NH_4)_6Mo_7O_{24} \cdot 4H_2O$, graphene oxide	800	2	Mo_2C	[19, 20]
	Template Assisted: MOF $H_3PMo_{12}O_{40}$ $[Cu_2(BTC)_4/3(H_2O)_2]_6[H_3PMo_{12}O_{40}]$	800-900	3	MoC_{1-x}	[21, 22]
**Tungsten Carbides (not ultrathin 2D materials)**	Reduction: WO_2 in CH_4–H_2	700~800	10	WC, β-W_2C	[23, 24, 25]
	CVD: Cu/W in CH_4–H_2; W in 1,1,3,3-tetramethyl-1,3-disilacyclobutane	1100~2400	1~4	WC, W_2C	[26]
	Template-assisted: graphitic C_3N_4 (mpg-C_3N_4) template + WCl_6	700~1000	1~5	WC, W_2C	[22, 27, 28]
	Solid Reaction: graphene nanoplatelets (GnP) + ammonium tungstate + aniline	900	3	W_2C	[29]
	Pyrolysis: $W(CO)_6$ + Mg powder	900	3	WC	[30]
	Wet Chemical: C + tungsten + H_2O_2	1000	24	W_2C	[31, 32]
**MXenes (ultrathin 2D)**	MAX Phase Reduction: MAX phase (Mo_2Ga_2C) with HF etching	1600	160	Mo_2C	[21, 33, 34]
**This work (ultrathin 2D)**	**Laser ablation on metallo-hydrogel**	**Ambient**	**ms**	**MoC_x, WC_x, CoC_x**	

Supplementary Notes 1

Spin coating curve. Gelatin coordinated with different transition metal shows different colors, such as the yellowish semi-transparent thin film spin-coated on glass using a Mo-gel solution (2m, 60 wt %). The calibration of the spin coating thickness curve, essential for reproducible roll-to-roll production is decided by the hydrogel viscosity and rotational speed, and we use a semi-empirical plot to fit the power law of the rotation speed[35].

Supplementary Notes 2

MoC_x. From XRD spectrum, the peaks are consistent with $MoC_{0.66}$ from the database which is a carbon deficient phase. The lattice constant slightly shrinks (4.26 A) as compared with that of the intrinsic α-MoC (4.36A), which is also supported by a simple DFT model (**Supplementary Figure 2**). In the model a 2×2×2 supercell is built, and different portion (1-x, $0<x<1$) of carbon are deleted from the cell pseudo-randomly to build a MoC_x cell. Carbon deficient cells are relaxed, and lattice parameters are averaged from different configurations. For the energy storage application, varying the carbon ratio won't change the double layer capacitance. Meanwhile, Li doesn't have strong intercalation with MoC. Changing the carbon ratio will likely change the Li diffusion in the electrode but not likely to affect the capacitance too much. DFT simulation of $MoC_{0.66}$ and MoC didn't reveal great difference of their absorption coefficients, while the interconnected pore structure has the bigger effect on the absorption. Carbon defects in MoC will decrease the thermal conductivity, which is in favor for thermal lose protection. However, the thermal conduction loss is negligible than the thermal loss in the open system. To be more accurate, we change the interpretation to MoC_x ($x<1$).

Supplementary Notes 3

Temperature Simulation

1. Model Equation: heat transfer in solid.

$$\rho C_p \frac{\partial T}{\partial t} + \rho C_p \vec{u} \cdot \nabla T + \nabla \cdot \vec{q} = Q + Q_{ted}$$
$$\vec{q} = -k\nabla T \quad ,$$
$$-\vec{n} \cdot \vec{q} = q_0$$

ρ is the density of material, C_p is the heat capacity, k is the thermal conductivity.

$$H_f(x, y, T) = P\_laser \times g(x) \times g(y)$$

With inward heat flux which is the absorbed laser power and $g(x) \times g(y)$ is the geometry dispersion.

2. Simulation setups

- Materials set up are listed in **Supplementary Table 1**
- Equivalent heat source: Gaussian Pulse $g(x) \times g(y)$, is Gaussian distribution
- Physics: Heat Transfer in Solids (including radiative transfer)
- Mesh type: Physical standard, **fine size from 2.0E-6 to 2.0E-5 m**
- Laser spot radius: 0.1mm (200μm in diameter)
- Cutting speed: neglect
- Emissivity: 0.8

Supplementary Notes 4

IR absorption of metallo-hydrogen. The Mo-gelatin composite has the highest IR absorption at the typical CO_2-laser wavelength at 10.6 μm comparing to other gelatin composites containing same concentration of other metal ions. Specifically, Mo^{5+} ions have the [Kr]4d1 ground state, of which the degeneracy of the d-orbital can induce a strong absorption band near 1000 cm^{-1}(10 μm)[36]. The W^{5+} ion with a same shell band structure can be reduced from W^{6+} also contributes strong absorption in the far-IR range. Experimental results show good absorption of the Co^{2+}-gelatin

complex in the far-IR range over the pure Co^{2+} ions probably due to the strong ligand-Co stretching and vibration in the matrix material[37].

Supplementary Notes 5

Freezing temperature depression[38]

$$\Delta T_F = K_F \cdot b \cdot i \sim 74K$$

- ΔT_F, the freezing-point depression, is defined as $T_{F\,(pure\ solvent)} - T_{F\,(solution)}$ (water is 0 °C)
- K_F, the cryoscopic constant, which is dependent on the properties of the solvent, not the solute. (Note: When conducting experiments, a higher K_F value makes it easier to observe larger drops in the freezing point. For water, K_F = 1.853 K·kg mol^{-1}.)
- b is the molality (moles solute per kilogram of solvent) (21M kg^{-1} H_2O)
- i is the van 't Hoff factor (number of ion particles per individual molecule of solute,

e.g. i = 2 for NaCl, 3 for $BaCl_2$). (LiTFSI i=2)

Supplementary Notes 6

Analysis of Water-transport through the porous carbide membrane

$P_{vis} = \frac{8\mu L v_{liq}}{r_p^{\,2}}$, Viscous loss along the pore is calculated using the Hagen-Poiseuille equation. μ is the dynamic viscosity of water (1.002 mPa·s at 20 °C).

$v_{liq} = \dot{q}'' / \rho_{liq}\Delta h_{lv}$. Bulk liquid velocity can be calculated from the energy balance with heat flux $\dot{q}''$ normalized by pore area.

$P_{cap} = \frac{2\gamma_{lv}\cos\theta}{r_p}$. Capillary pressure is calculated by Young-Laplace equation. During operation, the interface self-adjusts the θ to have enough capillary pressure (calculated by the Young-Laplace equation) to overcome the viscous loss. $\gamma_{lv} = 0.0728 Jm^{-2}$ at 20 °C.

Assume $\cos\theta = 0.5$, P_{cap} is three orders larger than the viscous loss which is expected in the experiment to avoid dry-out.

Supplementary Notes 7

Calculation of dielectric properties based on density functional perturbation theory. For the frequency-dependent dielectric function, $\varepsilon(\omega) = \varepsilon_1 + \mathrm{i}\,\varepsilon_2$, we employed the RPA approach with the local field effects included at Hartree level. RPA dielectric constants in GGA usually match well with experimental measurements for conventional semiconductors[39], although overshoots were also reported for transition-metal oxides due to band-gap underestimation with GGA. In our case, as shown in **Figure 3e**, the calculated optical transmission coefficient based on these RPA results coincide satisfactorily with our experimental data. This also justifies our calculations on the other optical properties shown in **Figure 3**, where the absorption coefficient α was calculated by

$$\alpha(\omega) = \frac{\sqrt{2}\omega}{c_0}\sqrt{-\varepsilon_1(\omega) + |\varepsilon(\omega)|}$$

where c_0 is light speed in vacuum, and $|\varepsilon| = \sqrt{\varepsilon_1^2 + \varepsilon_2^2}$ is the magnitude of the complex dielectric function. Similarly, the refractive index *n* and extinction coefficient *k* can be calculated as

$$n^2(\omega) = \frac{1}{2}\,(\varepsilon_1(\omega) + |\varepsilon(\omega)|)$$

and

$$k^2(\omega) = \frac{1}{2}\,(-\varepsilon_1(\omega) + |\varepsilon(\omega)|)$$

Notice that $\alpha(\omega) = 2\omega k(\omega)/c_0$.

Supplementary References

1. Sathish, C. I., Shirako, Y., Tsujimoto, Y., Feng, H. L., Sun, Y., Akaogi, M., Yamaura, K. Superconductivity of Delta-$MoC_{0.75}$ Synthesized at 17 Gpa, *Solid State Commun* **177**, 33-35 (2014).
2. Zang, X., Shen, C., Chu, Y., Li, B., Wei, M., Zhong, J., Sanghadasa, M., Lin, L. Laser Induced Molybdenum Carbide-Graphene Composites for 3D Foldable Paper Electronics, **30**, 1800062 (2018).
3. Winter, J., Shifler, D. The Material Properties of Gelatin Gels: MARVALAUD INC WESTMINSTER MD; 1975.
4. Kong, J.-Y., Miyawaki, O., Nakamura, K., Yano, T. The "Intrinsic" Thermal Conductivity of Some Wet Proteins in Relation to Their Hydrophobicity: Analysis on Gelatin Gel, *Agricult. Bio. Chem.* **46**, 783-788 (1982).
5. Xie, X., Li, D., Tsai, T.-H., Liu, J., Braun, P. V., Cahill, D. G. Thermal Conductivity, Heat Capacity, and Elastic Constants of Water-Soluble Polymers and Polymer Blends, *Macromolecules* **49**, 972-978 (2016).
6. Zeng, X. M., Martin, G. P., Marriott, C. Effects of Molecular Weight of Polyvinylpyrrolidone on the Glass Transition and Crystallization of Co-Lyophilized Sucrose, *Int. J. Pharm.* **218**, 63-73 (2001).
7. Meilleur, L., Hardy, A., Quirion, F. Probing the Structure of Pluronic Peo-Ppo-Peo Block Copolymer Solutions with Their Apparent Volume and Heat Capacity, *Langmuir* **12**, 4697-4703 (1996).
8. Chen, W., Gu, J., Liu, Q., Luo, R., Yao, L., Sun, B., Zhang, W., Su, H., Chen, B., Liu, P., Zhang, D. Quantum Dots of 1T Phase Transitional Metal Dichalcogenides Generated Via Electrochemical Li Intercalation, *ACS Nano* **12**, 308-316 (2017).
9. Lee, J. S., Oyama, S. T., Boudart, M. Molybdenum Carbide Catalysts: I. Synthesis of Unsupported Powders, *J. Catalysis* **106**, 125-133 (1987).
10. Zheng, W. Q., Cotter, T. P., Kaghazchi, P., Jacob, T., Frank, B., Schlichte, K., Zhang, W., Su, D. S., Schuth, F., Schlogl, R. Experimental and Theoretical Investigation of Molybdenum Carbide and Nitride as Catalysts for Ammonia Decomposition, *J. Am. Chem. Soc.* **135**, 3458-3464 (2013).
11. Wolden, C. A., Pickerell, A., Gawai, T., Parks, S., Hensley, J., Way, J. D. Synthesis of Beta-Mo2c Thin Films, *ACS Appl. Mater. Inter.* **3**, 517-521 (2011).
12. Xu, C., Wang, L., Liu, Z., Chen, L., Guo, J., Kang, N., Ma, X.-L., Cheng, H.-M., Ren, W. Large-Area High-Quality 2d Ultrathin Mo2c Superconducting Crystals, *Nat. Mater.* **14**, 1135-1141 (2015).
13. Gogotsi, Y. Chemical Vapour Deposition: Transition Metal Carbides Go 2D, *Nat. Mater.* **14**, 1079-1080 (2015).
14. Nagai, M., Shishikura, I., Omi, S. Molybdenum Carbide Prepared by Chemical Vapor Deposition, *Jpn J. Appl. Phys. 1.* **39**, 4528-4531 (2000).
15. Li, J. S., Wang, Y., Liu, C. H., Li, S. L., Wang, Y. G., Dong, L. Z., Dai, Z. H., Li, Y. F., Lan, Y. Q. Coupled Molybdenum Carbide and Reduced Graphene Oxide Electrocatalysts for Efficient Hydrogen Evolution, *Nat. Commun.* **7**, 11204 (2016).
16. Liao, L., Wang, S. N., Xiao, J. J., Bian, X. J., Zhang, Y. H., Scanlon, M. D., Hu, X. L., Tang, Y., Liu, B. H., Girault, H. H. A Nanoporous Molybdenum Carbide Nanowire as an Electrocatalyst for Hydrogen Evolution Reaction, *Energ. Environ. Sci.* **7**, 387-392 (2014).

17. Khabbaz, S., Honarbakhsh-Raouf, A., Ataie, A., Saghafi, M. Effect of Processing Parameters on the Mechanochemical Synthesis of Nanocrystalline Molybdenum Carbide, *Int. J. Refract. Met. H.* **41**, 402-407 (2013).
18. Kwak, W. J., Lau, K. C., Shin, C. D., Amine, K., Curtiss, L. A., Sun, Y. K. A Mo2c/Carbon Nanotube Composite Cathode for Lithium-Oxygen Batteries with High Energy Efficiency and Long Cycle Life, *Acs Nano* **9**, 4129-4137 (2015).
19. Chen, M. H., Zhang, J. W., Chen, Q. G., Qi, M. L., Xia, X. H. Construction of Reduced Graphene Oxide Supported Molybdenum Carbides Composite Electrode as High-Performance Anode Materials for Lithium Ion Batteries, *Mater. Res. Bull.* **73**, 459-464 (2016).
20. Ihsan, M., Wang, H. Q., Majid, S. R., Yang, J. P., Kennedy, S. J., Guo, Z. P., Liu, H. K. $MoO_2/Mo_2C/C$ Spheres as Anode Materials for Lithium Ion Batteries, *Carbon* **96**, 1200-1207 (2016).
21. Halim, J., Kota, S., Lukatskaya, M. R., Naguib, M., Zhao, M. Q., Moon, E. J., Pitock, J., Nanda, J., May, S. J., Gogotsi, Y., Barsoum, M. W. Synthesis and Characterization of 2d Molybdenum Carbide (Mxene), *Adv. Funct. Mater.* **26**, 3118-3127 (2016).
22. Zhu, J. X., Sakaushi, K., Clavel, G., Shalom, M., Antonietti, M., Fellinger, T. P. A General Salt-Templating Method to Fabricate Vertically Aligned Graphitic Carbon Nanosheets and Their Metal Carbide Hybrids for Superior Lithium Ion Batteries and Water Splitting, *J. Am. Chem. Soc.* **137**, 5480-5485 (2015).
23. Nagai, M., Kakinuma, T., Matsuda, K. Temperature-Programmed Reduction and Oxidation of Tungsten Carbide Catalyst for Low-Temperature Water-Gas Shift Reaction, *J. New Mat. Electr. Sys.* **10**, 217-220 (2007).
24. Ribeiro, F. H., Betta, R. a. D., Guskey, G. J., Boudart, M. Preparation and Surface-Composition of Tungsten Carbide Powders with High Specific Surface-Area, *Chem. Mater.* **3**, 805-812 (1991).
25. Hara, Y., Minami, N., Itagaki, H. Synthesis and Characterization of High-Surface Area Tungsten Carbides and Application to Electrocatalytic Hydrogen Oxidation, *Appl. Catal. a-Gen.* **323**, 86-93 (2007).
26. Shi, Y. J., Badran, I., Tkalych, A., Kan, W. H., Thangadurai, V. Growth of Crystalline Tungsten Carbides Using 1,1,3,3-Tetramethyl-1,3-Disilacyclobutane on a Heated Tungsten Filament, *J. Phys. Chem. C.* **117**, 3389-3395 (2013).
27. Gong, Q. F., Wang, Y., Hu, Q., Zhou, J. G., Feng, R. F., Duchesne, P. N., Zhang, P., Chen, F. J., Han, N., Li, Y. F., Jin, C. H., Li, Y. G., Lee, S. T. Ultrasmall and Phase-Pure W_2C Nanoparticles for Efficient Electrocatalytic and Photoelectrochemical Hydrogen Evolution, *Nat. Commun.* **7**, 13216 (2016).
28. Garcia-Esparza, A. T., Cha, D., Ou, Y. W., Kubota, J., Domen, K., Takanabe, K. Tungsten Carbide Nanoparticles as Efficient Cocatalysts for Photocatalytic Overall Water Splitting, *Chemsuschem* **6**, 168-181 (2013).
29. Chen, W. F., Schneider, J. M., Sasaki, K., Wang, C. H., Schneider, J., Iyer, S., Iyer, S., Zhu, Y. M., Muckerman, J. T., Fujita, E. Tungsten Carbide-Nitride on Graphene Nanoplatelets as a Durable Hydrogen Evolution Electrocatalyst, *Chemsuschem* **7**, 2414-2418 (2014).
30. Pol, S. V., Pol, V. G., Gedanken, A. Synthesis of WC Nanotubes, *Adv. Mater.* **18**, 2023-2027 (2006).
31. Meng, H., Shen, P. K. The Beneficial Effect of the Addition of Tungsten Carbides to Pt Catalysts on the Oxygen Electroreduction, *Chem. Commun.*, 4408-4410 (2005).

32. Tang, C. Y., Wang, D. Z., Wu, Z. Z., Duan, B. H. Tungsten Carbide Hollow Microspheres as Electrocatalyst and Platinum Support for Hydrogen Evolution Reaction, *Int J Hydrogen Energ* **40**, 3229-3237 (2015).
33. Naguib, M., Mashtalir, O., Carle, J., Presser, V., Lu, J., Hultman, L., Gogotsi, Y., Barsoum, M. W. Two-Dimensional Transition Metal Carbides, **6**, 1322-1331 (2012).
34. Mashtalir, O., Naguib, M., Mochalin, V. N., Dall'agnese, Y., Heon, M., Barsoum, M. W., Gogotsi, Y. Intercalation and Delamination of Layered Carbides and Carbonitrides, *Nat. Comm.* **4**, 2664 (2013).
35. Meyerhofer, D. Characteristics of Resist Films Produced by Spinning, *J Appl Phys* **49**, 3993-3997 (1978).
36. Reddy, S. L., Reddy, G. S., Endo, T. *Electronic (Absorption) Spectra of 3d Transition Metal Complexes*. INTECH Open Access Publisher, 2012.
37. Miller, F. A., Wilkins, C. H. Infrared Spectra and Characteristic Frequencies of Inorganic Ions - Their Use in Qualitative Analysis, *Anal. Chem.* **24**, 1253-1294 (1952).
38. Mellor, J. W. *Modern Inorganic Chemistry*. (Longmans, Green, 1912).
39. Shishkin, M., Kresse, G. Self-Consistent Gw Calculations for Semiconductors and Insulators, *Phys. Rev. B* **75**, 235102 (2007).
